# The Effects of Ginger on Fasting Blood Sugar, Hemoglobin A1c, Apolipoprotein B, Apolipoprotein A-I and Malondialdehyde in Type 2 Diabetic Patients

**Published:** 2015

**Authors:** Nafiseh Khandouzi, Farzad Shidfar, Asadollah Rajab, Tayebeh Rahideh, Payam Hosseini, Mohsen Mir Taheri

**Affiliations:** a *Department of Nutrition and Biochemistry, School of Nutritional Sciences and Dietetics, Tehran University of Medical Sciences, Tehran, Iran.*; b *Department of Nutrition, School of Public Health, Iran University of Medical Sciences, Tehran, Iran.*; c *Iranian Diabetes Association, Tehran, Iran.*; d *Department of Nutrition, School of Public Health, Iran University of Medical Sciences, Tehran, Iran. *; e *Razi Hospital-Psychiatry, Tehran, Iran. *; f *Razi Hospital-Psychiatry, Tehran, Iran.*

**Keywords:** Ginger, Glycemic status, Apolipoproteins, Malondialdehyde, Diabetes mellitus

## Abstract

Diabetes mellitus is the most common endocrine disorder, causes many complications such as micro- and macro-vascular diseases. Anti-diabetic, hypolipidemic and anti-oxidative properties of ginger have been noticed in several researches. The present study was conducted to investigate the effects of ginger on fasting blood sugar, Hemoglobin A1c, apolipoprotein B, apolipoprotein A-I, and malondialdehyde in type 2 diabetic patients. In a randomized, double-blind, placebo-controlled, clinical trial, a total of 41 type 2 diabetic patients randomly were assigned to ginger or placebo groups (22 in ginger group and 19 in control group), received 2 g/day of ginger powder supplement or lactose as placebo for 12 weeks. The serum concentrations of fasting blood sugar, Hemoglobin A1c, apolipoprotein B, apolipoprotein A-I and malondialdehyde were analyzed before and after the intervention. Ginger supplementation significantly reduced the levels of fasting blood sugar, hemoglobin A1c, apolipoprotein B, apolipoprotein B/apolipoprotein A-I and malondialdehyde in ginger group in comparison to baseline, as well as control group, while it increased the level of apolipoprotein A-I (p<0.05). It seems that oral administration of ginger powder supplement can improves fasting blood sugar, hemoglobin A1c, apolipoprotein B, apolipoprotein A-I, apolipoprotein B/apolipoprotein A-I and malondialdehyde in type 2 diabetic patients. So it may have a role in alleviating the risk of some chronic complications of diabetes.

## Introduction

Diabetes mellitus can be defined as a group of metabolic diseases characterized by chronic hyperglycemia resulting from impaired insulin action/secretion and is classified into two major categories, type 1 and type 2. Type 2 diabetes accounts for >90% of diabetes and is resulting in impaired function in carbohydrate, lipid and protein metabolism. Effective control of hyperglycemia in diabetic patients is critical for reducing the risk of micro- and macro-vascular diseases (1). 

The prevalence of diabetes mellitus has reached epidemic proportions and has affected 6.4% of adults worldwide in 2010 (2). The global prevalence for all age groups was estimated to be 4.4% in 2030 (3). The number of patients suffering from diabetes, among the 25-64 years old Iranians is 7.7%, equal to 2 million patients, which half of them are not aware of their disease. As well as, 6.8%, equal to 4.4 million of Iranian adults have impaired fasting glucose (4). 

Dyslipidemia (lipid abnormalities) resulting from uncontrolled hyperglycemia and insulin resistance in diabetic patients is a major risk factor for coronary artery disease, stroke and peripheral vascular disease (5). 

Recently, attention has been focused on the relationship between production of free radicals, especially reactive oxygen species (ROS), and the pathogenesis as well as progression of diabetes mellitus. Mechanisms that contribute to the formation of free radicals in diabetes mellitus may include metabolic stress resulting from changes in energy metabolism, inflammatory mediators and impaired antioxidant defense mechanisms (5). Hyperglycemia increases oxidative stress through the overproduction of reactive oxygen species, which results in an imbalance between free radicals and the antioxidant defense system of the cells (3). It was reported that oxidative stress which affects carbohydrate, lipid and protein metabolism (3), increase in patients with diabetes mellitus and suggested to induce endothelial cell dysfunction and trigger the progression of atherosclerosis (6). The elevated blood glucose levels in diabetes are thought to lead to cell death through oxidative stress induction (3). Because of the increased risk of cardiovascular disease in these patients, the preventive strategy of normal blood lipids and reduction of oxidative stress should be considered. 

Apart from conventional anti diabetic therapy, medicinal plants, complementary and alternative medicine therapies have beneficial effects and improve glucose homeostasis in diabetic patients (7). Several ethno pharmacological studies on medicinal plants having beneficial effects on diabetes have been reported (8). Ginger is an underground rhizome of plant *Zingiber officinale* belonging to the family Zingiberaceae and it is one of the most widely consumed spices worldwide. It has a long history of use as herbal medicine to treat a variety of diseases including nausea and vomiting, constipation, indigestion (dyspepsia), pain, and cold induced syndromes. More recently, it was reported that ginger also possesses anti-cancer, anti-clotting, anti-inflammatory, and anti-oxidative characteristics, since it can scavenge superoxide anion and hydroxyl radicals (2). 

Ginger is known to contain a number of potentially bioactive substances, mainly gingerols and their related dehydration products, the shogaols, as well as volatile oils including sesquiterpenes, such as beta-bisabolene and (-)-zingiberene, and monoterpenes, mainly geranial and neral (9). In addition, phytochemical reports have shown that the main constituents of ginger are gingerol, shogaol, zingerone and paradol. It was reported that 6-gingerol and 6-shogaol are the major gingerol and shogaol present in the rhizome (1). 

Ginger has been shown to possess anti-diabetic activity in a variety of studies. Akhani *et al.* (2004) reported that ginger pretreatment inhibited the induced hyperglycemia and hypoinsulinemia (10). Other investigators have showed the hypolipidemic effect of ginger (11). Other studies suggested that the response to ginger components depends on it's dose concentration (12,13). Some experimental researches published on anti-diabetic, hypo-lipidemic and anti-oxidative properties of ginger are controversial and more investigations may clarify it's potency in protection and treatment of metabolic disorders (6).

Therefore, the present study was planned to evaluate the effects of ginger powder supplementation on serum levels of fasting blood sugar (FBS), Hemoglobin A1c (HbA1c), apolipoprotein B (apo B), apolipoprotein A-I (apo A-I), Apo B/Apo A-I, and malondialdehyde (MDA) in type 2 diabetic patients.

## Experimental

*Materials and methods*

The present study was a randomized, double blind, placebo controlled clinical trial performed on type 2 diabetic patients. The population of the study included all adult patients, aged 20-60 years old, who were referred to the Iran diabetes association in Tehran. The under study patients were diagnosed with non-insulin dependent diabetes mellitus (NIDDM) by an endocrinologist on the basis of the results of the blood tests and met the criteria of the study. These criteria included: disease duration at least 2 years, HbA1c level of 6-8%, taking no antioxidant supplements such as selenium, zinc and beta-carotene for at least 3 months prior to the study, no smoking and drinking. Exclusion criteria of the study were insulin therapy at baseline or during the study, changes in the type or dose of medication, changes in diet or daily physical activity, any acute illnesses or some chronic diseases including kidney, liver, cardiovascular, and gastrointestinal diseases, smoking, pregnancy and lactation, consumption of ginger or other botanical supplements, ginger hypersensitivity, and consumption of less than 80% of supplements during the study period.

Sample size was determined based on data from previous study (14) by considering type I error level α=0.05 with test power of 80%. To insure that the sample size, used in the study, could obtain the desired outcome for all relevant variables, the highest number of the calculated samples was considered. The sample size was computed as 20 per group. Regarding a possible loss to follow-up, a safety margin of 25% was determined, and therefore 25 patients were allocated in each group.

After attending the orientation session and fulfilling written informed consent paper, the participants were enrolled, and 50 eligible patients of either sex included in the study. 

Patients were divided randomly into two groups (experiment and control, 25 subjects in each) using computer's random numbers to receive either ginger or placebo one capsule twice a day for 12 weeks (15). All subjects were permitted to consume their usual medications according to their physician's recommendation. At the beginning of the study, information on socio-economic status of participants was collected by means of a questionnaire completed through an interview. The report of daily dietary intake was collected by a 24-hour diet recall questionnaire once at the beginning and at the end of the study. The data was then analyzed by Nutritionist IV software. The reports of the level of physical activity, at the beginning and the end of the study, were obtained through interview with individuals using the International Physical Activity Questionnaire (IPAQ). The patients were instructed to maintain their diet and physical activity during the intervention. 

Anthropometric parameters including height and weight were measured at the beginning and the end of the intervention to calculate body mass index (BMI) as the formula (weight (Kg)/height squared (m^2^)). Body weight was measured without shoes and light clothing by using a seca scale with the precision of 0.5 Kg. Heights were also measured using a stadiometer without shoes with precision of 0.5 cm. During the 12-week intervention practice, the patients were called to resolve possible problems and ensure that the participants had consumed the drugs and supplements. 

Blood samples (10 mL) were taken in a 10-12 hours fasting state at the beginning and after 12 weeks of intervention. The serum was obtained by high speed centrifugation and was frozen immediately at −80 °C until assay. 

The concentration of FBS was measured by enzymatic method using eliteh kit (via Hitachi machine) from French company of Feppim 717. Measurement of serum HbA1c level was performed by turbidometric inhibition immunoassay method using Roche kit from Germany. Apo B and apo A-I were also assayed by turbidometric immunoassay method using Roche kits (via Cobas machine) from Germany. Serum level of MDA was calculated by spectrophotometry method (Satoh 1978) (16) according to the instruction.

Fresh rhizomes of *Zingiber officinale* were purchased from local market and were ground as a fine particle after drying. The powder was delivered to a pharmaceutical lab (Tehran university of medical sciences, Iran) to prepare capsules containing 1 gram ginger in each (17). Lactose was also used to make placebo (18). The capsules were placed in the identical bottles by a third person not directly involved in this study. This person labeled the bottles with 2 cods which retained unknown for researchers and patients until the end of intervention. To evaluate the compliance of patients, bottles containing unconsumed ginger (or placebo) capsules were given monthly, and if was >20% of total delivered supplements the patient was excluded from the intervention.

The data were analyzed by SPSS software (version 16) and the results were expressed as mean ± standard deviation (Mean ± SD). The normality of the distribution of variables was determined by the Kolmogorov-Smirnov test. The differences in the levels of blood biochemical variables were compared, before and after the intervention, in each group. For variables with normal distribution, a paired t-test and for variables other than those, a wilcoxon test was used. To compare quantitative variables between the two groups, independent t-test and Mann-withney test were used for normal and non normal distribution variables, respectively. Differences with p-value <0.05 were considered to be statistically significant. 

The protocol of study was in compliance with the Helsinki Convention of 1975 (revised in 2008) and informed consent form was obtained from each patient. The study was approved by the Ethics Committee of Public Health School of Tehran University of Medical Sciences (No. 2402), and registered in Iranian center of clinical trial registration with the ID number of IRCT 201109082709N19.

**Figure 1:**
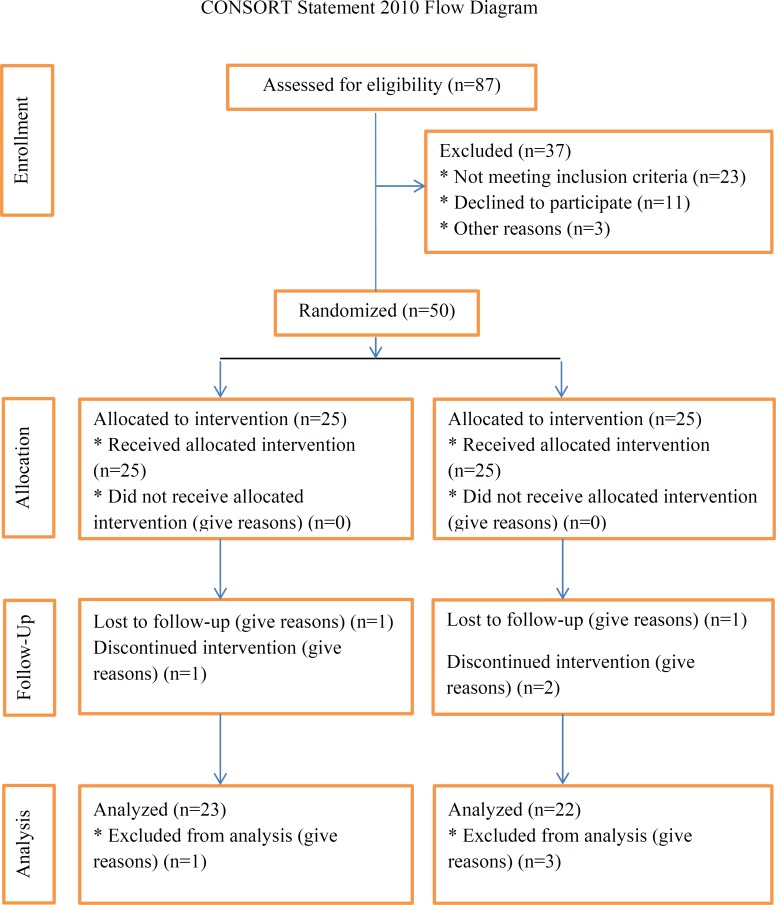
The consort flowchart describing the progress of the patients through the trial.

## Results

Of 50 patients initially recruited, 41 persons (22 in the ginger group and 19 in the control group) completed the study, and 9 persons were excluded because of migration, change in type and dose of medication, and refusing to continue. There were 5 males and 17 females in the ginger group with an average age of 45.20 ± 7.64 years old and 9 males and 10 females with an average age of 47.10 ± 8.31 years in the control group.

There were no significant differences between the intervention and control groups regarding the distribution of sex, age, weight, waist circumference, body mass index (BMI), disease duration, type of consumed oral hypoglycemic drugs, daily dietary intake and the level of physical activity, at the beginning and the end of the study (p>0.05).

The table showed that there were no statistically significant differences in the baseline levels of FBS, HbA1c, Apo B, Apo A-I and Apo B/Apo A-I between the ginger and placebo groups (p>0.05), while the level of MDA was statistically different (p = 0.014). 

Ginger supplementation significantly reduced the levels of FBS (p=0.000), HbA1c (p=0.000), Apo B (p=0.000), Apo B/Apo A-I (p=0.000) and MDA (p=0.001), as well as increased the level of Apo A-I (p=0.000) in the ginger group in comparison to the baseline. While the levels of FBS (p=0.048), Apo B (p=0.000), Apo B/Apo A-I (p=0.000) and MDA (p=0.004) increased, and Apo A-I (p=0.008) reduced in placebo group during the study. However, the statistical analysis indicated that the observed changes in control group may be the result of time effect, placebo empathic effect or other unknown factors, and it's not intervention dependent (Table 1). 

As well as, results exhibited statistically significant differences in mean difference values of FBS, HbA1c, Apo B, Apo A-I, Apo B/Apo A-I and MDA (p=0.0000) between two groups at the end of study (Table 1).

**Table 1:** The mean and standard deviation (Mean ± SD) of blood biochemical variables at the beginning and the end of the study.

variable	time	Groups		p-value^*^
		Ginger (n=22)	Placebo (n=19)	
FBS (mg/dl)	Before intervention	161.50 ± 58.01	155.47 ± 81.83	0.785
	After intervention	142.09 ± 47.90	157.10 ± 81.83	0.875
	Difference	-19.41 ± 18.83	1.63 ± 4.28	0.000
p-value**		0.000	0.048	_
HbA1c (%)	Before intervention	7.37 ± 1.86	7.30 ± 1.31	0.619
	After intervention	6.60 ± 1.26	7.32 ± 1.32	0.083
	Difference	-0.77 ± 0.88	0.02 ± 0.16	0.000
p-value^**^		0.000	0.513	_
Apo B (mg/dl)	Before intervention	92.95 ± 39.44	89.21 ± 20.67	0.701
	After intervention	80.50 ± 36.69	90.07 ± 20.48	0.302
	Difference	-12.45 ± 10.57	0.86 ± 0.57	0.000
p-value^**^		0.000	0.000	_
Apo A-I (mg/dl)	Before intervention	141.36 ± 68.43	119.55 ± 3.73	0.431
	After intervention	161.91 ± 71.73	118.85 ± 3.89	0.001
	Difference	20.54 ± 14.74	-0.70 ± 1.23	0.000
p-value^**^		0.000	0.008	_
Apo B/Apo A-I (mg/dl)	Before intervention	0.72 ± 0.29	0.75 ± 0.18	0.719
	After intervention	0.52 ± 0.17	0.76 ± 0.17	0.000
	Difference	-0.20 ± 0.16	0.01 ± 0.01	0.000
p-value^**^		0.000	0.000	_
MDA (nmol/mL)	Before intervention	3.75 ± 1.38	2.89 ± 0.66	0.014
	After intervention	2.90 ± 0.98	2.95 ± 0.69	0.637
	Difference	-0.85 ± 1.08	0.06 ± 0.08	0.000
p-value^**^		0.001	0.004	_

* independent t-test for normal distribution variables, and Mann-Withney test for non-normal distribution variables

** paired t-test for normal distribution variables, and wilcoxon test for non-normal distribution variables

## Discussion

The present study was conducted to investigate the effects of ginger on fasting blood sugar (FBS), Hemoglobin A1c (HbA1c), apolipoprotein B (Apo B), apolipoprotein A-I (Apo A-I), Apo B/Apo AI, and malondialdehyde (MDA) in type 2 diabetic patients.

In this study, it was demonstrated that oral administration of ginger powder for 12 weeks at dose of 2 g per day caused significant reduction in the levels of FBS, HbA1c, Apo B, Apo B/Apo A-I and MDA in ginger group in comparison to baseline, as well as control group, while it increased the level of Apo A-I.

Ojewole J.A.O. (2006) reported that oral intake of alcoholic extract of ginger (800 mg/Kg) significantly decreased the level of fasting blood sugar after 1 hour treatment in STZ-diabetic rats. The effect peak was observed after 4 hours and 24-53% reduction of blood glucose with consumption of doses of 100-800 mg/Kg (19). As well as, Islam M.S. & Choi H (2008), in nicotinamide and low dose STZ-diabetic rat model, noticed that oral administration of ginger powder at dose of 200 mg/Kg resulted in alleviation of metabolic syndrome signs including blood glucose and serum lipids reduction and increasing total antioxidant capacity (TAC) (20). Our results are similar to these results. However, Bordia A. *et al.* (1997) stated that the consumption of 4 g/day ginger powder in coronary artery disease (CAD) duration 3 months did not change the level of serum glucose and lipids (21). The differences in our results with this study may be due to difference in chemical composition of administered ginger extract, preparation method, rhizome used, or storage time (22,23). Jafri SA. *et al.* (2011) showed that oral administration of ginger extract with daily dose of 500 mg/Kg for 6 weeks in Alloxan-diabetic rats caused decreased in blood glucose level at 21 and 42 days (24). Abdulrazaq NB. *et al*. (2010) in a study found that daily administration of oral ginger aqueous at dose of 500 mg/Kg during 30 days in STZ-diabetic rats caused 38% and 68% reduction in plasma glucose level, on the 15^th^ and 30^th^ day of study, respectively. This solution have hypoglycemic effect by increased the activity of glycolytic enzymes (glucokinase, phosphofructokinase, pyruvate kinase) (25). Another study performed by Saeid JM. *et al*. (2010) indicated that administration of oral ginger extract in diabetic chickens for 6 weeks led to decreased the serum levels of glucose, triglyceride (TG), total cholesterol (TC), and LDL-C, and increased serum HDL-C level (26). Apo A-I as the main apolipoprotein of HDL structure is the serum level representative of HDL-C. Several studies found a strong relationship between the elevation of plasma HDL-C and Apo A-I, the change in plasma Apo A-I concentration explaining almost half (approximately 48%) of the variation in HDL-C concentration during the course of the intervention. This relationship could either reflect a decrease in the clearance of HDL-C particles and/or an increase in the synthesis of Apo A-I during the course of the intervention. As well as, apo B as the main apolipoprotein of LDL structure, is the serum level representative of LDL-C, and decrease of Apo B in this study means that small, dense LDL was significantly less than control group. However, apo B and apo A-I were better than LDL-C and HDL-C, respectively, in predicting cardiovascular risk in type 2 diabetes (27). Khadem Ansari MH*. et al*. (2008) found blood glucose concentration have more decreased in STZ-diabetic rats treated with ginger powder (5% of daily dietary intake for 6 weeks) compared to control diabetic rats. The HbAlc level in the ginger-treated group was significantly lower than that in the non treated diabetic group. It has been showed that HbAlc level is increased during diabetes and it is a marker which shows the degree of protein glycation. Administration of ginger to diabetic rats significantly decreased the level of glycosylated hemoglobin and this may be due to the decreased level of blood glucose (28). Our results are in agreement with these results. Many investigators reported that compounds of ginger such as 6-gingerol, tannins, polyphenolic compounds, flavonoids, and triterpenoids possess hypoglycemic and other pharmacological properties (3). Rani MP. *et al.* (2010) suggested that ginger, via it's major component, gingerol, by inhibition of key enzymes relevant to type 2 diabetes, *α*-glucosidase and α-amylase, are known to improve diabetes (29). Li Y. *et al*. (2012) found that polar portion of ginger extract containing mainly gingerols, particularly (S)-[6]- and (S)-[8]- gingerol, promoted glucose uptake significantly in cultured rat skeletal muscle cells. This action of gingerols was attributed to facilitation of insulin-independent glucose uptake by increasing translocation of glucose transporter GLUT4 to the muscle cell plasma membrane surface, together with small increases in total GLUT4 protein expression (1). Another mechanism for reducing blood glucose by ginger hydroalcoholic extract, is the inhibition of hepatic phosphorylase enzyme, hereby it prevents the breakdown of hepatic glycogen storages, also, can increases the activity of enzymes improving glycogen synthesis. The other possible effect is suppression the activity of hepatic "glucose 6-phosphatase" enzyme, that causes degradation of glucose 6-phosphate to glucose, and consequently increases blood glucose level (30). *In-vitro* studies suggested that ethyl acetate extract of ginger have inhibitory effect on the two key enzymes of glucose metabolism (α-amylase and α-glucosidase); the function of ginger against these two enzymes was found to be correlated with phenolic content of gingerol and shogaol at these extracts. Ginger has been shown to modulate insulin release. Ginger promotes glucose clearances in insulin responsive peripheral tissues, which is crucial in maintaining blood glucose homeostasis (29). As well as, it is reported that 6-gingerol increases the glucose uptake at insulin responsive adipocytes (31). Thus, at treated cells with gingerol, insulin responsive glucose uptake has increased and improved diabetes (30). Several studies stated that ginger have permanent effects of reducing lipids, and accordingly, increases insulin sensitivity. Owing to some studies showed that increasing the level of plasma free fatty acids (FFAs) lead to insulin resistance, ginger indicate anti-insulin resistance effects by reducing the level of plasma FFAs (32). Elshater A.A.E. *et al*. (2009) indicated that daily oral administration of ginger extract (4 mg/Kg) to STZ-diabetic rats resulted in reducing the plasma level of glucose, total cholesterol, LDL-C, and increasing HDL-C, comparing to control rats (2). The hypocholesterolemic effects of ginger may be due to the inhibition of cellular cholesterol synthesis, since, ((E)-8 beta, 17-epoxyllabed-12-ene-15, 16 dial) compound was isolated from ginger and interfered with cholesterol biosynthesis in liver homogenate in hypercholesterolemic mice causing it's reduction (33). Shanmugam K.R. *et al*. (2009) noticed that feeding with a diet containing 1% and 2% ginger powder during 30 days period decreased serum level of glucose and malondialdehyde (MDA) in STZ-diabetic rats, while it didn’t influence in normal rats (3). Moreover, Madkor H.R*. et al*. (2011) indicated that addition of ginger (1%) to a normal diet prevented the formation of free radicals and maintained the integrity of rat erythrocytes. The antioxidant potency of ginger has been attributed to gingerols that prevent reactive oxygen species (ROS) production (5). More than 50 antioxidants have been isolated from ginger rhizome; these antioxidants include two groups of components related to gingerols and diarylheptanoids (34). The major pungent component of ginger is gingerol, a mixture of homologues with 10, 12 and 14 carbons in the side chain designated (6)- (8)- and (10)-gingerols. Gingerols can be converted to shogoals and zingerone by dehydration and retro-aldol reaction, respectively (7). In one study, ginger extract consumption inhibited LDL oxidation in apolipoprotein-E deficient mice; this could be due to direct ability of ginger extract in scavenging free radicals (35). Harliansyah A.H. *et al.* (2005) indicated that the treatment of human normal liver cells and liver cancer cells with 500 µg/mL of ginger extract, *in-vitro*, didn’t change the content of malondialdehyde (MDA); our findings are inconsistent with these findings. However, ginger contains three main compounds of gingerol, shogaol, and paradol, which exhibit antioxidant activity (36). Al-Azhary D.B. (2011) stated that oral administration of ginger extract with daily dose of 25 mg/Kg for 6 weeks in STZ-diabetic rats, according to our finding, caused significant reduction in blood glucose, triglyceride, and malondialdehyde, and increasing in plasma total antioxidant capacity (TAC), while it couldn’t return increased total cholesterol and LDL-C to normal level. Ginger has been reported to have a lowering effect on lipid peroxidation, thus MDA level, by influencing the enzymatic blood level of superoxide dismutase (SOD), catalase (CAT), and glutathione peroxidase (GPX). It has been also shown that ginger reduces cellular oxidation and scavenges superoxide anion and hydroxyl radicals (6). Lebda M.A. *et al*. (2012) suggested that intake of different forms of ginger (powder, warm or cold extract) in amount 2% of basal diet in rabbits resulted in significant decline in serum level of TG, TC, LDL-C, MDA, while it increased the level of blood glucose and HDL-C. Reduction of lipid peroxidation by ginger has been attributed to it's antioxidant activity, because ginger have many phenolic compounds, which have inhibitory effects on lipid peroxidation and preserve the antioxidant compounds (33). The basic concentration of the serum glucose of the rabbits in that study was lower than that of our study patients, the rabbits were not diabetic; as well as, the comparison between that study findings with our finding was impossible, because it may be different the glucose metabolism in humans and rabbits body. 

In conclusion, the present study showed that ginger supplementation significantly reduced the levels of FBS, HbA1c, Apo B, Apo B/Apo A-I and MDA, and increased the level of Apo A-I in type 2 diabetic patients. Regarding negligible side effects of ginger, it may be a good remedy for diabetic patients to diminish the risk of some secondary chronic complications. 

The present study was the first human research investigating the effects of ginger on glycemic status and stress oxidative in type 2 diabetic patients whose information regarding potential confounding variables, including dietary intake and physical activity level were available. However, this study had limitations, as well. First, several patients participating in the intervention, were excluded from the study. Moreover, frequent inclusion criteria, especially having no metabolic diseases and taking no antioxidant supplements, led to further reduction in the number of patients eligible for the study and hampered the disease finding process with many difficulties.

Finally, it is suggested to perform similar studies with higher number of patients and longer study period for a better observation of the effects of ginger in improving diabetic patient status.
